# Recent Insights into the Role of DNA Methylation and Histone Modifications in Systemic Sclerosis: A Scoping Review

**DOI:** 10.3390/diagnostics14060652

**Published:** 2024-03-20

**Authors:** Tsvetelina Kostova, Rositsa Karalilova, Zguro Batalov, Maria Kazakova, Victoria Sarafian, Anastas Batalov

**Affiliations:** 1 Department of Propedeutics of Internal Diseases, Medical University of Plovdiv, 4000 Plovdiv, Bulgaria; 2 Clinic of Rheumatology, UMHAT Kaspela, 4000 Plovdiv, Bulgaria; 3 Department of Medical Biology, Medical University of Plovdiv, 4000 Plovdiv, Bulgaria; 4 Research Institute at Medical University of Plovdiv, 4000 Plovdiv, Bulgaria

**Keywords:** systemic sclerosis, epigenetics, DNA methylation, histone modifications

## Abstract

Systemic sclerosis is a complex idiopathic disease originating from an intricate interplay between genetic susceptibility, environmental factors, and epigenetic modifications. This scoping review aims to map the advancements made regarding DNA methylation abnormalities and histone modifications in systemic sclerosis in the past decade. A literature search was conducted using three electronic databases (Scopus, Web of Science and PubMed) to identify relevant articles. A total of 44 studies were selected for this review, demonstrating the critical contribution of epigenetic perturbations in multiple cell types to disease pathogenesis. In conclusion, this scoping review has elucidated the significant discoveries made in the past decade regarding the role of DNA methylation and histone modifications in systemic sclerosis. Further progress in the field could lead to the development of novel treatment possibilities targeting epigenetic marks.

## 1. Introduction

Systemic sclerosis (SSc) is an idiopathic complex immune-mediated disease characterized by microangiopathy and immune dysregulation leading to multiorgan fibrosis with complex interplay between multiple cell types [1]. It is widely acknowledged that a pivotal event in SSc pathogenesis is microvascular injury with endothelial cell activation. The progression of vascular damage leads to a reduction in the number of capillaries with thickening of vessel walls, which ultimately results in the narrowing of their lumen [2]. Inflammation and immune cell activation play a crucial role in SSc pathogenesis with dysregulation observed in both innate and adaptive immunity. These have been considered to result in tissue fibrosis, which is the distinguishing hallmark of SSc [2,3]. 

Despite the vigorous research and substantial progress in understanding the etiopathogenesis of SSc, it remains elusive. The results from genomic studies, including candidate-gene and genome-wide association studies have shown susceptibility loci associated with SSc within and outside of the HLA region [4,5]. However, they are indicative of a general autoimmunity and have been found in other autoimmune diseases, and, thus, are not specific for SSc [6]. Furthermore, it has been shown that SSc occurs significantly more frequently in families with scleroderma (1.6%) compared to the general population (0.026%) [7], yet the concordance rate for disease in monozygotic twins is only 4.7% [8]. This indicates that genetic susceptibility is of a substantial significance, although it is insufficient to solely explain disease development [6]. 

Studies have shown that immune cells and fibroblasts from patients with SSc maintain their activated phenotype for days after being taken out of their original environment despite the lack of a major underlying genetic influence [6]. The current hypothesis is that this is the result of the interaction between environmental factors and the cellular epigenome, the latter being shown to coordinate gene expression [9]. By integrating both genetic and environmental factors, the epigenetic regulome provides a molecular mechanism linking genetic background and environmental exposures to the development of the disease [10]. 

Epigenetics refers to the heritable chemical modification of DNA and histones that affect gene expression without altering the nucleotide sequence [11]. There are three main mechanisms by which the epigenome exerts its regulatory effects: DNA methylation, histone posttranslational modifications (PTMs) and non-coding RNAs (ncRNAs). DNA methylation is the first identified and most widely studied epigenetic mechanism. It is the process of addition of a methyl group to the C5 position of the cytosine nucleotide, transforming it into 5-methylcytosine (5mC). This process is catalyzed by a family of enzymes, called DNA methyltransferases (DNMT). There are three DNMT members in mammals: DNMT1, DNMT3A and DNMT3B, each carrying their subtle functional differences [3,12]. DNA methylation is mainly associated with gene repression, since it can directly inhibit the binding of transcription factors to DNA or it can lead to recruiting proteins involved in gene silencing [12]. 

Histone modifications are another epigenetic mechanism of great importance that has been well studied. Chromatin consists of DNA wrapped around nucleosomes, which are histone octamers formed by two copies of each of the histones H2A, H2B, H3, and H4. The chemical changes in these histone proteins are known as posttranslational modifications. PTMs encompass a multitude of reversible modifications of the N-terminal tails of the histones, including acetylation, methylation, phosphorylation, lactylation and others [13]. They regulate gene expression through their ability to remodel chromatin, the latter being a dynamic structure that governs the access of transcriptional machinery to DNA. A plethora of enzymes are responsible for the maintenance of the histone landscape, namely through the addition of modifications to specific residues (“writers”), their interpretation (“readers”) and removal (“erasers”). Histone modifications can be related to either gene activation or repression [3,11,13].

NcRNAs are promising factors that can affect gene expression and modulate epigenetic evidence. These molecules require a better understanding of the molecular mechanisms and their alterations in the disease [14]. 

The field of epigenetics is rapidly evolving in the search for new pharmaceutical targets for SSc, and there is a substantial accumulation of literature demonstrating the importance of epigenetic aberrations in the effector cells. The aim of this scoping review is to map the research conducted in the last ten years on DNA methylation and PTMs in SSc. We sought to answer the following research question: “*What aberrancies in DNA methylation and PTMs in SSc have been discovered in the last decade and what is their role in the disease’s pathogenesis?”*

## 2. Methods

### 2.1. Search Strategy

We conducted a scoping review in accordance with the PRISMA extension for scoping review guidelines [15]. A three-step search strategy was utilized as recommended by JBI [16]. The first step included a thorough literature search using three electronic databases, including MEDLINE (PubMed), Web of Science and Scopus, performed in December 2023. We employed a search strategy using the terms (“*systemic sclerosis*”) *AND* (“*epigenetics*”). These terms allowed us to search for all studies pertaining to epigenetics and SSc ensuring that no relevant study would be missed from the database search. A filter for publishing date was placed in all databases to include articles in the time period from 2013 to 2023 (in Scopus 01-01-2013 to 01-12-2023). This was employed in order to ensure that only recent studies were going to be included in the current scoping review. Using the keywords, the search yielded a total of 542 articles across the three electronic databases. 

### 2.2. Eligibility and Selection of Studies

**Figure 1:**
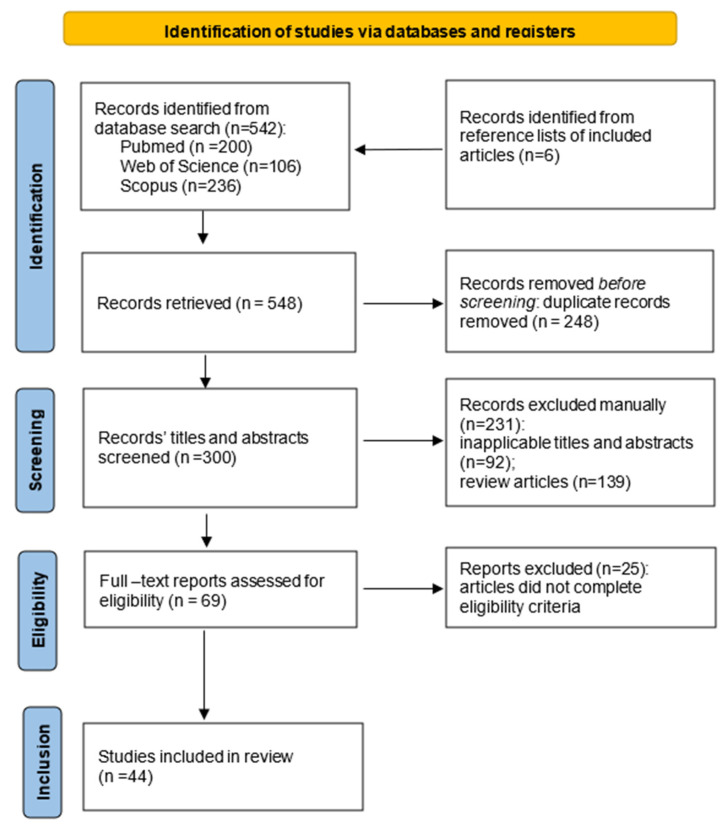
PRISMA flow diagram showing the process of identification, screening, and inclusion of studies.

The titles and abstracts from all retrieved records were screened for eligible studies. Articles with inappropriate titles and abstracts not pertinent to the research question and review articles were excluded. All full-text studies were screened for eligibility based on inclusion and exclusion criteria formulated by us. We included the following types of papers: (1) original articles containing information about DNA methylation aberrancies in all cell types; (2) original articles containing information about chromatin structure, and/or histone PTMs, and/or names of histone-modifying enzymes; (3) studies focused only on SSc, using isolated cells from patients and/or animal models—in vitro and in vivo studies; and (4) articles only in English. It is possible to have relevant studies missed due to this constraint. Studies were excluded if: (1) they analyzed epigenetic mechanisms in SSc together with other autoimmune diseases; (2) they were focused on localized scleroderma and not SSc; (3) they analyzed ncRNAs, including microRNAs and long non-coding RNAs; (4) they were published outside of the chosen time period from 2013 to 2023 (Figure 1).

### 2.3. Data Charting

A data-charting form was developed by two reviews to establish what information to retrieve from the included articles. The following relevant characteristics were extracted independently by two reviewers: (1) the names of authors; (2) the year of publication; (3) the aim of the study; (4) methods applied to analyze the epigenetic mechanisms; (5) cell type (model) used; (6) results; and (7) the importance of the findings pertaining to the pathogenesis of SSc. The form was continuously updated by the two reviewers, and the results and relevance of the findings were discussed jointly.

## 3. Results

### 3.1. Selection of Sources

The initial database search yielded 542 results. All records were added to a citation manager software and prior to screening, duplicates were removed, thus, leaving 295. Since the abstracts and titles of 92 of the retrieved articles were not pertaining to SSc and/or DNA methylation and histone PTMs, they were excluded. Another 139 review articles and letters to the editor were also removed. The remaining 69 full-text articles were assessed for eligibility based on the predefined inclusion and exclusion criteria, and of them, 25 articles were excluded because they did not meet the criteria—the research was conducted in multiple autoimmune diseases simultaneously and did not contain sufficient information on SSc, or they were not analyzing DNA methylation and histone PTMs. A total of 39 articles met the eligibility criteria and were included in the scoping review. Additionally, we manually screened the reference lists of the included articles and identified 6 relevant titles. After full-text assessment, 1 was excluded, thus, leaving 44 articles to be included in the final review (Figure 1). 

### 3.2. Characteristics of Sources and Results from Individual Sources 

The included articles were grouped based on the type of epigenetic mechanism they analyzed. The first group covers studies evaluating DNA methylation abnormalities, comprising whole genome-wide methylation and targeted methylation analyses. The second group contains studies analyzing chromatin architecture, histone PTMs and histone-modifying enzymes (‘‘writers’’ and ‘’erasers’’). There is one study that reports both DNA methylation aberrancies and histone PTMs, and is, thus, included in both groups. The studied tissue type, methods of evaluation of the epigenetic mechanisms, main results, and significance of the findings for both groups are presented in Table 1 and in the Supplementary Table S1.

### 3.3. Synthesis of Results

Most of the included studies conducted between 2013 and 2023 from both groups focused on the research of the epigenetic mechanisms in human dermal fibroblasts (FBs). From the studies performed on adaptive immune cells, seven analyzed human CD4+ T-cells, one of which used combined data from CD4+ and CD8+ T-cells and one used human B-cells. Of the investigations conducted on cells of innate immunity, three focused on monocytes, one on macrophages and two on dendritic cells (DCs). Five studies were performed in peripheral blood mononuclear cells (PBMCs) and only two studies used whole blood specimens. There were five reports performed on microvascular endothelial cells (MVECs). There are two studies that employed a study population of twins discordant for SSc.

### 3.4. New Insights into DNA Methylation and Role in Pathogenesis

In the current scoping review, eleven publications analyzed DNA methylation in SSc FBs. Two studies performed a global genome-wide DNA methylation assay and demonstrated global hypomethylation of SSc FBs [35,36]. Furthermore, Altorok et al. uncovered that the two disease subsets, limited cutaneous SSc (lcSSc) and diffuse cutaneous SSc (dcSSc) displayed distinct methylation patterns with a small number of shared differentially methylated CpGs. This study also found a significant overexpression of the majority of hypomethylated genes (*ITGA9*, *ADAM12*, *COL23A1*, *COL4A2* and *RUNX3*) involved in pathways relevant to disease pathogenesis that are shared between lcSSc and dcSSc. Moreover, the authors identified hypomethylation of *CTNNA2* and *CTNNB1* in dcSSc and *CTNNA3* and *CTNND2* in lcSSc [35]. 

Additionally, Hattori et al. established that active demethylation has a role in SSc. This study confirmed the global hypomethylation status in SSc FBs, associated with mRNA overexpression of a demethylating agent from the ten-eleven translocation family (TET), TET1, which converts 5mC to 5-hydroxymethylcytosine (5hmC) [37]. 

In contrast, targeted analyses established the hypermethylation status of genes crucial for SSc pathogenesis. A 2020 study reported hypermethylation at the suppressor of the cytokine signaling 3 (SOCS3) promoter as a result of the TGF-β-mediated induction of DNMT3A and DNMT1 [38]. SOCS3 is a regulator of JAK/STAT signaling and its epigenetic repression stimulated fibroblast-to-myofibroblast transition, collagen release and fibrosis in vivo and in vitro [38]. Another study reports altered methylation of the promoter regions of friend leukemia integration 1 (Fli-1) and Krüppel-like factor 5 (KLF5), which led to their downregulation in SSc FBs [19]. KLF5 works synergistically with Fli1 in suppressing connective tissue growth factor (CTGF) transcription, which is a key fibrotic mediator. The authors suggest that the downregulation of both transcription factors induces the three major manifestations of SSc [19]. Furthermore, hypermethylation was found at the promoters of genes encoding for Dickkopf WNT signaling pathway inhibitor 1 (DKK1) and secreted frizzled-related protein 1 (SFRP1) [39]. The latter are known antagonists of Wnt, and their downregulation led to pathological activation of canonical Wnt/β-catenin signaling. Zhang et al.’s research discovered hypermethylation at the promoter of the poly(ADP-ribose) polymerase-1 (PARP-1) gene associated with decreased expression of PARP-1. PARP-1 was found to negatively regulate TGF-β and its epigenetic silencing could contribute to enhanced TGF-β signaling and persistent fibroblast activation [40].

Of interest, two studies examined methyl CpG-binding protein 2 (MeCP2), a known methyl-binding protein (MBD) in SSc FBs with conflicting results [41,42]. He et al. established that MeCP2 inhibited myofibroblast differentiation, migration, and proliferation by directly binding to regulatory sequences in *NID2* and *PLAU* gene loci [41]. In contrast, Henderson et al. suggested that MeCP2 epigenetically repressed SFRP1, which led to enhanced Wnt signaling [42]. 

#### 3.4.1. In Immune Cells

Six studies employed a genome-wide DNA methylation assay, seven performed targeted analysis and one used a whole genome hydroxymethylation assay in immune cells.

Widespread differential methylation was established in CD4+ T-cells from patients with SSc for genes involved in pathways such as Wnt/β-catenin and Hippo signaling, which have been shown to be involved in fibrosis [43]. A large-scale analysis, published in 2018 revealed predominant hypomethylation and the upregulation of type I interferon (IFN)-associated genes in both CD4+ and CD8+ T-cells, along with an increase in circulating IFN protein levels. This suggests that DNA methylation aberrations partly underlie the upregulation of type I IFN and contribute to the immune dysregulation observed in SSc [44]. 

The results of a 2020 study indicate an effect of DNA methylation on gene expression through long-range enhancer interactions involving CCC-TC-binding factor (CTCF) in CD4+ T-cells [45].

Targeted analysis identified the hypomethylation of the promoter of *ITGAL* in CD4+ T-cells, which encodes CD11a, which was significantly correlated with CD11a overexpression [46]. This could contribute to immune dysregulation since CD11a is a cell-surface molecule, which is essential for T-cell co-stimulation and the initiation of immune responses. Additionally, a study found hypermethylation at the forkhead box protein (FOXP3) promoter, which contributes to its reduced expression in CD4+ T-cells [47]. This transcription factor is important for the normal development and the suppressive capacity of T-regs. The reduced expression of FOXP3 is suggested to be responsible for the decreased numbers of T-regs, which may further mediate the immune dysfunction in SSc [47].

An important study by Zeng et al. published in 2022 found that DNA demethylation has a role in the abnormal activation of CD4+ T-cells in SSc [48]. The authors identified that 2′-5′ oligoadenylate synthase (OASL), a key antiviral factor induced by IFNs, mediates global hydroxymethylation by the upregulation of TET1 expression. This enhances CD40L and CD70 expression levels and induces the aberrant activation of CD4+ T-cells [48]. 

We identified only one study to analyze isolated monocytes from SSc patients [49]. The results from this study in which the authors analyzed a population of African American patients with SSc using a genome-wide DNA methylation assay demonstrated modest DNA methylation and gene expression differences between SSc patients and healthy controls.

The analysis of DNA methylation in a population of plasmacytoid dendritic cells (pDC) was found in only one study [50]. pDC is a specialized subpopulation of dendritic cells (DCs) which serves as antigen-presenting cells and are capable of producing type I IFN upon activation. The authors found hypermethylation at the *RUNX3* gene, which encodes runt-related transcription factor 3 (RUNX3). The latter is known to contribute to the differentiation and regulation of DCs. This hypermethylation was correlated with the downregulation of RUNX3, leading to impaired pDCs functionality in mouse models [50].

We identified five studies using a diverse population of peripheral blood mononuclear cells (PBMCs) showing abnormal DNA methylation that could contribute to immune dysfunction [39,51,52,53,54].

#### 3.4.2. In Endothelial Cells 

So far, only two studies have analyzed DNA methylation in SSc microvascular endothelial cells (MVECs) [55,56]. The first study by Wang et al. was published in 2013 and demonstrates the hypermethylation of the promoter of bone morphogenic receptor II (BMPRII), which correlated with the reduced expression of BMPRII. Bone morphogenic proteins regulate cell proliferation, differentiation and survival [56]. The reduced expression of BMPRII was correlated with decreased MVECs survival, and the addition of DNMT and HDAC inhibitors restored the expression levels. The second study by Nada et al., published in 2022, performed an unbiased genome-wide DNA methylation assay in SSc MVECs. The authors demonstrated the global hypomethylation of MVECs and further highlighted aberrancies in DNA methylation correlating with the expression of specific genes involved in SSc pathogenesis, namely *ANGPT-2*, *NOS1*, *DNMT3A*, *DNMT3B* and *HDAC4* [55]. 

### 3.5. New Insights into Histone PTMs, Associated Enzymes and Chromatin Landscape and Role in Pathogenesis

#### 3.5.1. In FBs

Most of the studies analyzing histone PTMs, “writers” and “erasers”, and the chromatin landscape conducted between 2013 and 2023 focused on FBs. A 2019 study assessing chromatin accessibility and transcriptome profiling discovered the constitutive activation of a newly found TGFB2 enhancer through epigenetic memory [18]. The resulting TGFβ2 signaling maintains a profibrotic state in ex vivo FBs. The authors show that the enhancer exhibits epigenetic marks of enhancer activity—elevated acetylation of H3K27 (H3K27ac) and occupancy by the histone acetyltransferase (HAT) p300. An earlier study from 2013 assessed the same “writer” p300 in SSc FBs and found it to be upregulated in lesional skin biopsies. They provide evidence for its induction by TGF-β, and further show that TGF-β enhanced both p300 recruitment and in vivo histone H4 acetylation at the *COL1A2* (collagen, type I, a2) gene, thus, p300 participates in fibrogenesis [23]. A study by Zehender et al. implicates MYST1, a HAT that mediates acetylation on histone H4 lysine 16 in the control of autophagy in SSc [30]. MYST1 regulates the expression of components, which participate in autophagy. The authors show that TGF-β induces autophagy by downregulating MYST1, which results in collagen release and induces tissue fibrosis. 

Histone deacetylation has also been found to contribute to the pathogenesis of SSc in FBs. In the study by Noda et al., in addition to the altered methylation status at the promoter of KLF5 and Fli1, the authors also identified the hypoacetylation of H3 and H4 at the same locations. This further contributes to the downregulation of KLF5 and Fli1, which demonstrates two different epigenetic mechanisms leading to their repression [19]. Moreover, two studies analyzed sirtuin 1 (SIRT1), which is a class III histone deacetylase (HDAC) in SSc FBs and reported discrepancies regarding its role in the pathogenesis of SSc [25,26]. The first study provided evidence in support of potent antifibrotic effects exerted by SIRT1 [25]. In contrast, the other report suggests it amplifies TGF-β signaling and fibrosis [26]. A 2015 study provides an additional role for HDAC in fibrosis [34]. The authors demonstrate that persistently active TGF-β signaling uses HDAC-mediated mechanisms to inhibit NR4A1, an endogenous inhibitor of TGF-β [34].

Studies conducted in the selected time frame also analyzed the effect of histone methylation and demethylation in FBs. Enhancer of zeste homologue 2 (EZH2) catalyzes the trimethylation at lysine 27 on histone H3 (H3K27me3), which is known to repress the transcription of target genes. EZH2 was overexpressed in FBs and endothelial cells (ECs) in a study by Tsou et al. and was found to promote fibrosis by stimulating cell migration, gel contraction and profibrotic genes [20]. Furthermore, EZH2 inhibits angiogenesis through repressing the Notch signaling pathway [20]. In contrast, while Krämer et al. also report increased levels of H3K27me3 in FBs, they suggest that this mark acts as a negative regulator of fibrosis [21]. It exerts its antifibrotic effects by repressing Fos-related antigen 2 (Fra-2), a transcription factor shown to regulate the release of collagen from FBs. We identified a single report on the role of the histone demethylase Jumonji domain-containing protein D3 (JMJD3), which catalyzes the demethylation of H3K27me3 in SSc FBs [27]. The authors suggest it exerts its profibrotic action by reducing the levels of the inhibitory histone mark H3K27me3 at the promoter of *FOSL2*, a gene encoding the profibrotic Fra-2.

A recent large-scale analysis performed by Tsou et al. reported the global reduction in chromatin accessibility in FBs and ECs in SSc and identified neural pathways that could contribute to angiogenesis and fibrosis [28]. A previous study in dermal ECs in SSc demonstrated the overexpression of HDAC5 that contributes to impaired angiogenesis in SSc by repressing pro-angiogenic factors [29].

#### 3.5.2. Immune Cells

An important study in 2020 created an epigenetic regulome of eight resident cell types in the affected and unaffected skin of SSc patients [17]. This study demonstrates the greatest disease-associated changes in chromatin accessibility in DCs, providing evidence for their contribution to disease pathogenesis.

A study identified genomic loci with aberrant H3K4me3 and H3k27ac marks in SSc monocytes [22]. The expression of 381 genes was found to be correlated with the presence of chromatin marks near their transcription start sites. Analysis revealed that the genes correlating with these histone marks were enriched for immune, IFN and cytokine signaling pathways [22]. 

A report of human CD4+ T-cells identified a global reduction in the gene repressive mark H3K27me3 [32]. Wang et al. analyzed SSc B-cells and demonstrated histone H4 hyperacetylation and a decrease in H3K9m3, both of which could lead to active transcription [31].

## 4. Discussion 

SSc has three hallmarks: microangiopathy, immune system dysregulation and tissue fibrosis [1]. In this scoping review we identified 44 original studies conducted between 2013 and 2023 analyzing DNA methylation aberrations, histone PTMs and the chromatin landscape in multiple cell types, involved in SSc pathogenesis. 

Vascular injury is an initiating and propagating factor in disease pathogenesis, and microangiopathy is one of the three hallmarks of SSc [57]. Considering this, studies in the field of SSc epigenetic research have assessed DNA methylation, histone-modifying enzymes, and the chromatin landscape in MVECs. DNA methylation aberrancies in genes relevant to the disease’s pathogenesis were established at the genome-wide level, as well as in targeted analyses [3,55,56]. Two histone-modifying enzymes, EZH2 and HDAC5, were found to be overexpressed in dcSSc and contributing to the impaired angiogenesis through different mechanisms [20,29]. 

Activation of the immune system has a central role in the pathophysiology of SSc with the dysregulation of both innate and adaptive immunity branches [2,3]. A study by Lei et al. in 2009 reported hypomethylation of CD4+ T-cells and a reduction in DNMT1 levels in patients with SSc compared to healthy controls [58]. Following this report, a targeted study established the hypomethylation of the promoter region of *CD40L* in female SSc patients with increased expression of CD40L, which plays a major role in the initiation of immune responses [59]. Similarly, a separate study established the hypomethylation of the promoter of *CD70* associated with the overexpression of CD70, a B-cell co-stimulatory molecule that promotes the differentiation of plasma cells and the synthesis of antibodies [60]. The overexpression of both molecules could be contributing to the dysregulation of the immune system observed in SSc.

The studies in the past ten years further improved the understanding of the intricate link between epigenetic mechanisms and immune abnormalities in SSc [3,6]. Widespread differential methylation was observed in CD4+ T-cells with aberrancies in genes involved in pathways integral to the disease’s pathogenesis [43,44,45,61]. The presence of a type IIFN signature has been well-established in SSc [50], and recent evidence has been suggesting that DNA methylation abnormalities could be contributing to the upregulation of type I IFN signaling [44]. Moreover, the discovery that the overexpressed OASL in CD4+ T-cells from SSc patients could upregulate TET1 implicates active demethylation to be contributing to the established hypomethylation of CD4+ T-cells and the overexpression of CD40 and CD70 in these cells [48]. In addition, histone marks associated with increased gene expression have been reported in CD 4+ T-cells and B-cells from SSc patients [31,32].

Additionally, this scoping review identified studies that discovered epigenetic alterations in cells of the innate immune system, including monocytes [22,49], DCs and pDCs [17,50], which highlight their role in SSc pathogenesis. Studies on PTMs in monocytes discovered altered chromatin marks that correlate with the IFN signature observed in these cells. Furthermore, histone modifications were implicated in the release of profibrotic molecules in SSc monocytes upon toll-like receptor 8 (TLR8) stimulation [3,24].

FBs and myofibroblasts are the central mediators in the development of tissue fibrosis, the most prominent hallmark of SSc [62]. FBs remain persistently activated and are responsible for the excessive production of collagen and other ECM components [62]. SSc FBs were found to be hypomethylated, which could be contributing to their pathological phenotype [35,37]. Furthermore, lcSSc and dcSSc were found to exhibit distinct methylation profiles with only a 6% overlap of differentially methylated CpGs between the two subsets, which could be contributing to their clinical heterogeneity [35]. Most of the shared CpGs were hypomethylated and in genes encoding for genes involved in TGF-β and Wnt-pathways. 

TGF-β signaling plays a crucial role in FBs’ activation and myofibroblast differentiation [62]. Dysregulated epigenetic mechanisms which are implicated in TGF-β signaling were reported. These include the discovery of a constitutively activated TGFB2 enhancer which was epigenetically maintained [18], the epigenetic silencing of SOCS3 and PARP-1 [38,40], and the downregulation of SIRT1 and MYST1, which contribute to enhanced TFG-β signaling [25,30].

Epigenetic alterations in transcription factors were also reported. Wang et al. first established the critical role of the collagen suppressor Fli-1, which was found to be downregulated by two epigenetic mechanisms, FLI1 promoter hypermethylation and histone H3 and H4 hypoacetylation [63]. In a separate study, Fli-1 was found to work synergistically with another transcription factor, KLF-5, in suppressing CTGF transcription [19]. This study showed that the epigenetic silencing of KLF-5 is also orchestrated by two different mechanisms, DNA methylation and histone hypoacetylation at the promoter of *KLF5* gene [19]. Fra2 is a profibrotic transcription factor, found to be controlled by JMJD3, which regulates the level of the repressive histone mark H3K27me [27]. Pharmacological inhibition of JMJD3 led to the downregulation of *FRA2* due to the induction of H3K27me3 marks at its promoter. A previous study by Krämer et al. reported that the inhibition of EZH2 exacerbated fibrosis due to the induction of Fra-2 [21]. In contrast to this, Tsou et al. report potent profibrotic effects of EZH2 [20].

Wnt signaling pathway is widely accepted as a central profibrotic pathway in SSc [62]. Activation of canonical Wnt signaling is dependent on the equilibrium between Wnt ligands and Wnt antagonists. In this scoping review, we identified studies that report the hypermethylation and underexpression of the Wnt antagonists DKK1 and SFRP1 [39], and the suppression of SFRP1 by MeCP2 [42]. Moreover, the hypomethylation of genes in the Wnt/β-catenin pathway such as *CTNNA3*, *CTNNB1* in dcSSc, and *CTNNA3* and *CTNND2* in lcSSc were found [35]. In contrast to the profibrotic properties of MeCP2, reported by Henderson et al., He et al. suggest that MeCP2 is antifibrotic [41]. The reason for this discrepancy is unknown, but it could be attributed to the diverse methodology applied in both studies and the heterogeneity of the studied population.

In summary, both explored epigenetic mechanisms in this scoping review shed light on the intricate and complex pathogenesis of SSc.

### Limitations

We conducted the literature search using three major electronic databases, excluding searches for difficult-to-locate or unpublished literature. Furthermore, we searched only for articles in English. Consequently, additional relevant studies might have been missed due to these constraints. A further limitation of this review is that we did not perform quality assessment of included publications, as our goal was to map out the existing literature related to the research question.

## 5. Conclusions

In conclusion, our scoping review has elucidated the significant discoveries made in the past decade regarding the role of DNA methylation and histone PTMs in SSc. These epigenetic alterations contribute to the vasculopathy, immune dysregulation and fibrotic hallmarks of SSc. Identifying pathways consistently altered by epigenetic disturbances is of considerable importance because of the potential for the therapeutic reversal of these abnormalities. Advancements in epigenetic research could lay the foundation for personalized medicine and introduce novel treatment possibilities targeting epigenetic modifications.

## Figures and Tables

**Table 1:** Characteristics of studies analyzing chromatin landscape and histone PTMs.

Cell Type/Model	Method	Results	Conclusion	Authors, Year
Clinically affected and unaffected SSc skin (8 resident cell types)	ATAC-seq	Significantly more differential peaks in DCs than the other resident cells. DCs display the most upregulated receptor/ligand interactions with other cell types. SSc-associated SNPs are predominantly enriched in DCs.	DCs possess the greatest disease-associated changes in chromatin accessibility.	Liu et al., 2020 [17]
Human dermal FBs	ATAC-seq	Higher accessibility at one of the enhancers of *TGFB2* with a correlation between the extent of chromatin accessibility and TGFB2 mRNA expression. The enhancer exhibits epigenetic marks—H3K27ac and occupancy by EP300 (enhancer activity). Inhibition of NF-kB or BRD4 achieved sustained inhibition of TGFB2 enhancer activity and mitigated pro-fibrotic gene expression.	Activation of a newly found enhancer of TGFB2 maintains a profibrotic state and is regulated epigenetically.	Shin et al., 2019 [18]
Human dermal FBs; murine models	ChIP assay	Hypoacetylation of H3 and H4 on the *KLF5* promoter. KLF5 and Fli1 synergistically repress CTGF transcription. Simultaneous downregulation of both KLF5 and Fli1 is a hallmark of SSc	Epigenetic downregulation of the antifibrotic factor KLF5.	Noda et al., 2014 [19]
Human dermal FBs and ECs; murine models	ChIP-seq, addition of DZNep, GSK126	Increased levels of EZH2 and H3K27me3. DZNep dose-dependently decreased EZH2, H3K27me3 and profibrotic genes. Overexpression of EZH2 stimulates cell migration, gel contraction and profibrotic genes. EZH2 inhibits angiogenesis by repressing the Notch signaling pathway. Enrichment of EZH2 binding and H3K27me3 marks at the promoter region of DLL4 in EC.	EZH2 is a key epigenetic factor that promotes fibrosis and inhibits angiogenesis in SSc.	Tsou et al., 2019 [20]
Human dermal FBs, murine models	Addition of DZNep, RT-PCR, Western blot, immunohistochemistry	Increased levels of H3K27me3. Inhibition of H3K27me3 with DZNep stimulates the release of collagen. DZNep exacerbates experimental fibrosis. Inhibition of H3K27me3 exerts its profibrotic effects by induction of FRA-2.	H3K27me3 acts as a negative regulator of tissue fibrosis by repressing the expression of FRA-2.	Krämer et al., 2013 [21]
Monocytes	ChIP-seq of H3K4me3 and H3K27ac	1046 and 534 genomic loci have aberrant H3K4me3 and H3K27ac marks. Gene expression significantly correlates and is proportional to the levels of these chromatin marks near gene transcription start sites. Upregulated genes are enriched in monocyte activation, IFN response and cytokine signaling pathways. Strong enrichment of binding sites for STAT and IRF TFs in the hypermethylated and hyperacetylated regions.	Alterations of the chromatin landscape impacting the transcriptome and gene expression of monocytes, correlating with their IFN signature.	Van der Kroef et al., 2019 [22]
Human dermal FBs	Immunostaining, semi-quantitative PCR, Western blot, immunofluorescence, ChIP	TGF-β stimulates the transcription of the HAT p300, thus, leading to its overexpression. This is independent of Smads and involves Egr-1. TGF-β leads to p300-dependent histone H4 hyperacetylation at the COL1A2 locus.	Histone acetylation mediated by p300 is an important epigenetic mechanism in fibrogenesis.	Ghosh et al., 2013 [23]
Human dermal FBs, human monocytes; murine models	ChIP assay	Fra-2 overexpression in skin biopsy samples from SSc patients and bleomycin-treated mice. TIMP-1 overexpression is induced by TLR-8 and mediated via Fra-2. Treatment with DZNep and the addition of TLR-8 agonist significantly increases Fra-2 and TIMP-1 expression in monocytes and induces transdifferentiation of FBs to myofibroblasts.	Epigenetic changes induced by DZNep have a role in TIMP-1 production mediated by Fra-2 in monocytes.	Ciechomska et al., 2016 [24]
Human dermal FBs, murine models	ChIP assay	Underexpression of SIRT1 in skin biopsy samples and in FBs. Activation of SIRT1 significantly attenuates the TGFβ-induced stimulation of FB contractility and migration. SIRT1 blocked Smad-dependent responses partly by the downregulation of the HAT p300 in explanted dermal FBs.	SIRT1 is underexpressed and it exerts potent antifibrotic effects by blocking Smad-dependent transcription.	Wei et al., 2015 [25]
Human dermal FBs, animal models	RT-PCR, western blot and immunohistochemistry	SIRT1 is downregulated in fibrotic skin mediated by enhanced TGF-β activation. SIRT1 activation stimulates TGF-β-induced FB activation and the release of collagen. Effective inactivation of SIRT1 in FBs exerts potent antifibrotic effects in murine models of experimental fibrosis.	SIRT1 is a positive regulator of TGF-β/Smad signaling. Downregulation of SIRT1 by TGF-β acts as an endogenous negative feedback mechanism to decrease TGF-β signaling in FBs.	Zerr et al., 2016 [26]
Human dermal FBs, murine models	ChIP assay	TGF-β-dependent overexpression of JMJD3 in SSc skin and in experimental fibrosis. JMJD3 promotes FB activation via FRA2. Inactivation of JMJD3 reverses the activated phenotype of FBs and promotes the accumulation of H3K27me3 at the FRA2 promoter, thus, reducing its expression. Pharmacological inhibition of JMJD3 ameliorated experimental fibrosis.	JMJD3 is profibrotic and modulates FB activation by regulating the levels of H3K27me3 at the promoter of FRA2.	Bergmann et al., 2017 [27]
Human dermal FBs and ECs	ATAC-seq	Chromatin accessibility is broadly decreased. Identification of differentially accessible chromatin loci enriched in pathways involved in the nervous system, cell membrane projections, cilia mobility, nitric oxide, and others. Increased chromatin binding of SNAI2, ETV2 and ELF1 in ECs, RUNX1 and RUNX2 in FBs. Upregulation of SNAI2 and ETV2 affects angiogenesis in ECs, the downregulation of ENTPD1 affects the profibrotic properties of FBs.	Global reduction in chromatin accessibility in ECs and FBs in dcSSc. Pathways related to neurons might play a role in the dysregulated angiogenesis and fibrosis.	Tsou et al., 2021 [28]
Dermal ECs	ATAC-seq	HDAC5 is overexpressed. HDAC5 knockdown increased tube formation in SSc ECs. ATAC-seq after HDAC5 knockdown identifies HDAC-5 regulated genes involved in angiogenesis and fibrosis (*FSTL1*, *PVRL2* and *CYR61*). HDAC5 mediates its anti-angiogenic effects partly by modulating chromatin accessibility.	Increased expression of HDAC5 represses several pro-angiogenic factors contributing to impaired angiogenesis in SSc.	Tsou et al., 2016 [29]
Human dermal FBs, murine models	Inhibition by TSA, inhibition of HMT	TGF-β promotes the activation of autophagy mediated by canonical TGF-β/SMAD3 signaling and repression of the H4K16 HAT MYST1. The latter regulates ATG7 and BECLIN1. Activation of autophagy stimulates FBt activation and induces fibrosis. Overexpression of MYST1 re-establishes the epigenetic control of autophagy.	The epigenetic control of autophagy is altered by a TGF-β dependent downregulation of MYST1.	Zehender et al., 2021 [30]
Human B-cells	Global histone H3/H4 acetylation and H3K4/H3K9 methylation assay	Global histone H4 hyperacetylation with global histone H3K9 hypomethylation. HDAC2, HDAC7 and SUV39H2 are significantly downregulated in contrast to JHDM2A which is upregulated. Global histone H4 acetylation was positively correlated with SDAI.	Global histone H4 hyperacetylation associated with disease severity and significant changes in the expression of genes that regulate histone acetylation.	Wang et al., 2013 [31]
Human CD4+ T-cells	Colorimetric H3K27 quantification assay	Global H3K27me3 levels are significantly lower with an inverse correlation with the levels of JMJD3. No differences between the expression levels of UTX, EZH1 and EZH2.	Global reduction in a gene-repressive mark.	Wang et al., 2015 [32]
Macrophages in lung samples	ScATAC-seq	Increased number of subpopulation of macrophages with the upregulated expression of SPP1 and MMP9. Increased accessibility of SPP1 and MMP9 in SPP1-macrophages. FABP4 gene shows more accessible chromatin in FABPR-macrophages. Transcription binding sites enriched in open chromatin identify multiple TFs: ATF5, TFEB, BCL11A, ETV5, JUN and others.	Identification of transcription factors in the profibrotic macrophages.	Papazoglou et al., 2022 [33]
Human dermal FBs, murine models	ChIP assay	Increased levels of NR4A1 in fibrotic skin. Short-term stimulation with TGF-β upregulates NR4A1 mediated by Smad signaling and the TF SP1. NR4A1 recruits the SP1-SIN3A-CoREST-LSD1-HDAC1 complex to reduce the expression of TGF-β target genes. Exposure of FBs to TGF-β for prolonged periods results in rapidly declining levels of NR4A1 mRNA and pan-NR4A1 protein. Rapid acetylation of histones H3 and H4 at the NR4A1 promoter upon TGF-β stimulation. Incubation with selective HDAC I and II inhibitors demonstrated that the desensitization of NR4A1 transcription is dependent on HDAC4, HDAC5, HDAC7 and HDAC10.	The persistently active TGF-β signaling uses HDAC-mediated epigenetic repression and AKT-induced phosphorylation to inhibit the NR4A1 negative feedback loop.	Palumbo-Zerr et al., 2015 [34]

ATAC-seq, assay for transposase-accessible chromatin with sequencing; DCs, dendritic cells; SNPs, single nucleotide polymorphisms; FBs, fibroblasts; ChIP, chromatin immunoprecipitation; KLF5, Krüppel-like factor 5; Fli1, friend leukemia integration-1; CTGF, connective tissue growth factor; EZH2, enhancer of zeste homolog 2; DLL4, delta-like canonical Notch ligand 4; RT-PCR, reverse transcription polymerase chain reaction; DZNep, 3-Deazaneplanocin; FRA-2, Fos-related antigen 2; IFN, interferon; STAT, signal transducer and activator of transcription; TF, transcription factor; HAT, histone acetyltransferase; TIMP-1, TIMP metallopeptidase inhibitor 1; TLR-8, toll-like receptor 8; SIRT1, sirtuin 1; JMJD3, Jumonji domain-containing protein D3; SNAI2, Snail family transcriptional repressor 2; ETV2, ETS variant transcription factor 2; 1; ECs, endothelial cells; HDAC, histone deacetylases; HMT, histone methyltransferase; ATG7, autophagy-related protein 7; UTX, ubiquitously transcribed tetratricopeptide repeat, X chromosome; NR4A1, nuclear receptor subfamily 4 group A member 1.

## Data Availability

Not applicable.

## Supplementary Materials

The following supporting information can be downloaded at https://www.mdpi.com/article/10.3390/diagnostics14060652/s1, Supplementary Table S1: Characteristics of the studies analyzing DNA methylation aberrancies in SSc. References [64,65,66] are cited only in Supplementary Table S1. References [19,29,35,36,37,38,39,40,41,42,43,44,45,46,47,48,49,50,51,52,53,54,55,56,61,64,65,66] are cited in the main text and in Supplementary Table S1.
