# Supplementary material for: Recent Insights into the Role of DNA Methylation and Histone Modifications in Systemic Sclerosis: A Scoping Review
Source: Diagnostics (Basel). 2024 Mar 20;14(6):652. doi: 10.3390/diagnostics14060652 (PMC10969595; doi:10.3390/diagnostics14060652)
Supplement: Supplementary file 1 [file diagnostics-14-00652-s001.zip › diagnostics-2897831-supplementary.pdf]

Table S1. Characteristics of the studies analyzing DNA methylation aberrancies in SSc.

Cell type/Model	Method for evaluation of DNA methylation	Results	Conclusion	Authors, year
Human dermal FBs from twins discordant for SSc	Genome-wide DNA methylation profiling and RNAseq	A total of 35 DMGs, predominantly hypomethylated. *HOXB3* and *TFAP2A* are overexpressed and hypermethylated, *HOXB8, HOXC10* and *TBX5* are overexpressed and hypomethylated. The overexpressed TFAP2A and TBX5 negatively regulate KLF4.	KLF4 is an anti-fibrotic factor that is downregulated early in disease progression, leading to fibroblast activation and fibrosis.	**Malaab et al., 2022** [64]
Human monocytes	Genome-wide DNA methylation	A total of 19 differentially methylated CpGs are identified in genes. Gene ontology analysis revealed that these genes participate in metabolic processes and there is a weak upregulation of immune pathways.	Modest DNA methylation and gene expression differences in monocytes.	**Allen et al., 2023** [49]
Human pDC, murine models	PCR-based TF profiling, methylation status analysis	Hypermethylation of the *RUNX3* gene, associated with a SNP. RUNX3 expression is increased after treatment with 5-Aza. Deletion of *RUNX3* in DCs leads to spontaneous induction of skin inflammation and fibrosis in untreated mice and increased severity of experimental fibrosis.	Epigenetic downregulation of RUNX3 alters the functionality and tissue distribution of pDCs in mice.	**Affandi et al., 2019** [50]
Human dermal FBs and human PBMCs; murine models	Methylation-specific PCR	Decreased expression of DKK1 and SFRP1 with hypermethylation of their promoters in FBs and PBMCs.	Epigenetic repression of the antifibrotic Wnt-antagonists DKK1 and SFRP1.	**Dees et al., 2014** [39]
Human CD4+ T-cells	Whole-genome bisulfite sequencing	A total of 340 DMGs are identified. Functional analysis showed their association with signaling pathways HIPPO, Wnt/β-catenin, RhoGDI, Netrin and Ephrin receptor signaling. A total of 12 DMGs on X-chromosome.	Widespread differential methylation of genes relevant to disease pathogenesis.	**Lu et al., 2019** [43]
Human dermal FBs	Genome-wide methylation	3528 differentially methylated CpGs (2710 in dcSSc, 1021 in	Distinct and characteristic	**Altorok at al., 2014** [35]

		lcSSc), only 6% are common between the two disease subsets, most of them being hypomethylated. Most genes that were hypomethylated in both disease subtypes (*ADAM12, COL23A1, COL4A2, ITGA9, MYO1E, PAX9 RUNX3* and *RUNX2*) are overexpressed.	methylation patterns in dcSSc and lcSSc. DNA methylation aberrancies associated with overexpression of genes in pathways crucial for fibrogenesis.	
Human MVECs	Genome-wide DNA methylation	Global hypomethylation. Differential methylation of genes–hypermethylated genes*: NOS1, DNMT3A, DNMT3B, HDAC4,* and *ANGPT2*. Hypomethylated: *IL17RA, CTNNA3, ICAM2,* and *SDK1*. Significant inverse correlation between DNA methylation status and gene expression. Decreased expression of ANGPT2, PDGFA, NOS1 and others.	A role of DNA methylation aberrancies in MVECs' dysfunction and angiogenesis.	**Nada et al., 2022** [55]
Whole blood from twins discordant for SSc	Genome-wide DNA methylation	A total of 153 unique cytosines in lcSSc and 266 distinct sites in dcSSc have differential methylation levels. 1% overlap of molecules shared between the two disease subsets. Consistent results between monozygotic and dizygotic twins.	Distinct DNA methylation pattern in disease subsets.	**Ramos et al., 2019** [65]
Human CD4+ T-cells	Bisulfite sequencing	Hypomethylation of CD11a promoter and increased expression of CD11a mRNA. CD11a affects the proliferative response of CD4+ T-cells to autologous PBMCs and induces IgG production by autologous B-cells. CD11a overexpressed on CD4+ T cells induces COL1A2 mRNA expression by normal FBs.	Epigenetic overexpression of CD11a may contribute to immune dysregulation and fibrosis.	**Wang et al., 2014** [46]
Human dermal FBs	Global methylation status analysis by LUMA	Overexpression of TET1 is associated with global hypomethylation. Hypoxia upregulates TET1 mRNA. Elevated expression of DNMT1 and DNMT3B.	Global hypomethylation associated with overexpression of a demethylating agent.	**Hattori et al., 2015** [37]
Human CD4+ T-cells	Bisulfite sequencing	*FOXP3* promoter hypermethylation associated with	Epigenetic downregulation of	**Wang et al., 2014** [47]

		downregulation of FOXP3. SDAI of patients with SSc was positively correlated with *FOXP3* promoter methylation and inversely correlated with FOXP3 mRNA expression.	FOXP3 may be responsible for quantitative defects in Tregs and immune dysregulation.	
Human CD4+ and CD8+ T-cells	Whole-genome DNA methylation microarray and targeted bisulfite sequencing	Differentially methylated sites in CD4+ and CD8+ T-cells. They have 215 genes in common with differentially methylated sites compared to controls. Global hypomethylation status of type I IFN-associated genes (*IFI44L, IFITM1, MX1, PARP9*) is shared in both CD4+ and CD8+ T-cells with upregulation of the genes.	Epigenetic aberrancies in the type I IFN pathway with upregulation of the associated genes.	**Ding et al., 2018** [44]
Human dermal FBs, murine models	Addition of 5-aza-20-deoxycytidine	KLF5 and Fli1 synergistically repress CTGF transcription. Simultaneous downregulation of both KLF5 and Fli1 is a hallmark of SSc. Treated FBs with 5-aza-20-deoxycytidine have an increase in KLF5 expression.	Epigenetic downregulation of the antifibrotic factor KLF5.	**Noda et al., 2014** [19]
Human CD4+ T-cells	DNA methylation analysis MethylationEPIC BeadChip array	Widespread changes in DNA methylation and the transcriptome.<br>DMPs and DEGs are enriched in regions related to inflammation and T cell biology. Pairs of DMP-DEG also form part of promoter-enhancer networks, potentially involving CTCF. Identification of SSc-associated susceptibility loci - *TNIP1* (rs3792783), *GSDMB* (rs9303277), *IL12RB1* (rs2305743), and *CSK* (rs1378942).	DNA methylation influenced gene expression through long-range enhancer interaction involving CTCF.	**Li et al., 2020** [45]
Human dermal FBs, murine models	Methylation-specific PCR, methylated DNA immunoprecipitation	SMAD3-dependent hypermethylation of SOCS3 promoter by TGF-β via upregulation of DNMT1 and DNMT3A, but not DNMT3B. Inhibition of epigenetic silencing of SOCS3 ameliorates experimental fibrosis.	Epigenetic silencing of SOCS3 as a result of chronic activation of TGF-β signaling.	**Dees et al., 2020** [38]

Human CD4+ T-cells	Whole Genomic DNA Hydroxymethylation assay	OASL overexpression could upregulate TET1 via IRF1 signaling activation, hence increase DNA hydroxymethylation levels. This leads to overexpression of CD40L and CD70.	OASL contributes to the regulation of abnormal hypomethylation of CD4+ T-cells and aberrant cell activation.	**Zeng et al., 2022** [48]
Human MVECs	Sequence analysis of DNA methylation; DNA bisulfite modification	Reduction of the expression of BMPRII correlated with promoter hypermethylation. Oxidation may trigger apoptosis in cells that underexpress BMPRII.	Epigenetic downregulation of BMPRII which has a role in preventing oxidation-induced apoptosis.	**Wang et al., 2013** [56]
Human PBMCs	Global DNA methylation analysis	Low overlap between DEGs and DMGs. A total of 20 differentially methylated and expressed genes with an inverse correlation between DNA methylation and gene expression. They are enriched in pathways related to immune cell migration, proliferation, activation, and inflammation.	Abnormal DNA methylation may contribute to sustained activation of PBMCs.	**Zhu et al., 2018** [51]
Human whole blood specimens	Absolute quantification of 5-mC, 5-hmdC, 5-cadC, 5-fdC, and 5-hmdU using 2D-UPLC-MS/MS	5-hmdU was significantly higher in SSc patients while 5-hmdC was lower compared to the HCs. 5-cadC and 5-fdC had upward trend in SSc.	Global hypomethylation pattern.	**Dal-Bekar et al., 2022** [66]
Human PBMCs	Methylation analysis by pyrosequencing	Hypomethylation of CpG sites in the enhancer region of T-reg specific *FOXP3*. No changes in methylation levels of the promoter. Imbalance of Treg cell subsets and abnormalities in TGF-β and IL-10 cytokine production by Treg cell subsets.	Upregulation of FOXP3 gene expression in active SSc suggest that epigenetic mechanism in part may underlie the imbalance of Treg cell function in SSc.	**Ugor et al., 2017** [52]
Human CD4+ T-cells	Whole-genome bisulfite sequencing, SOMNiBUS	131 differentially methylated regions and 125 DMGs.	Epigenetic alterations of genes known to be of interest in the pathogenesis using a novel computational method.	**Yu et al., 2023** [61]

Human dermal FBs	Reduced representation bisulfite sequencing	1180 differentially methylated CpGs. 17 genes with significant differential methylation, mostly in non-coding RNA genes and pseudogenes. Gene set enrichment and gene ontology analysis reveals an enrichment of pathways related to interferon signaling and mesenchymal differentiation.	Widespread DNA hypomethylation in African American patients with SSc.	**Baker Frost et al., 2021** [36]
Human PBMCs	PCR products of bisulfite-treated DNA sequencing	Insignificant promoter hypomethylation of *IRF7*. Overexpression of IRF7 mRNA in lcSSc, but not in dcSSc.	Hypomethylation of IRF7 promoter might promote IRF7 upregulation.	**Rezaei et al., 2017** [53]
Human PBMCs	Bisulfite pyrosequencing	Significant relative hypermethylation at the gene level for RORC1 and RORC2 in dcSSc, hypomethylation of FOXP3.	Epigenetic regulation of TFs.	**Almanzar et al., 2016** [54]
Human dermal FBs	ChIP-seq	Elevated expression of MeCP2 in dcSSc. Effects of MeCP2 are mediated, partially, through modulating *PLAU, NID2* and *ADA*. MeCP2 directly binds regulatory sequences in NID2 and PLAU gene loci.	MeCP2 is a novel antifibrotic epigenetic factor with inhibitory effects on myofibroblast differentiation, fibroblast migration and proliferation.	**He et al., 2018** [41]
Human dermal FBs	Immunoblotting, siRNA, lentiviral overexpression	Overexpression of MeCP2. TGF-β induces the expression of MeCP2 in normal cells. MeCP2 repressess the Wnt antagonist sFRP-1.	MeCP2 is a key epigenetic regulator and enhances Wnt signaling.	**Henderson et al., 2018** [42]
Human dermal FBs	Treatment with DZNep or PBS, MethylationEPIC BeadChip Array	37 differentially methylated CpG sites, corresponding to 11 hypomethylated and 13 hypermethylated genes. Some of them are relevant to fibrosis or fibrosis-related processess and decreased significantly after treatment with DZNep compared with PBS controls. DNMT1, DNMT3A and MeCP2 also decreased after DZNep treatment.	EZH2 affects the expression of DNMT1, DNMT3A and MeCP2 resulting in genome-wide methylation changes after treatment with DZNep.	**Tsou et al., 2019** [29]

Human dermal FBs, murine models	MeDIP and ChIP assays	Decreased expression of PARP-1 due to TGF-β-induced promoter hypermethylation. Inhibition of PARP-1 enhanced the effect of TGF-β on collagen release and myofibroblast differentiation in vitro.	PARP-1 is a negative regulator of TGF-β signaling and is silenced by promoter hypermethylation	**Zhang et al., 2018** [40]

DMG, differentially methylated genes; KLF4, Kruppel-like factor 4; RUNX, runt-related transcription factor, pDCs, plasmocytoid dendritic cells; FBs, fibroblasts; TF, transforming factor; TGF-β, transforming growth factor β; DKK1, Dickkopf WNT signaling pathway inhibitor 1; SFRP1, Secreted rizzled-related protein 1; PBMC, peripheral blood mononuclear cells; MVEC, microvascular endothelial cells; TET1, ten eleven translocation 1; DNMT, DNA methyltransferase; CTCF, CCCTC-binding factor; Fli-1, Friend leukemia integration 1 transcription factor; DMP, differentially methylated positions; DEG, differentially expressed genes; SOCS3, suppressor of cytokine signaling 3; OASL, 2-5-oligoadenylate synthetase like; IRF, interferon-regulatory factor; BMPRII, bone morphogenetic protein receptor type II; 2D-UPLC-MS/MS, Two-dimensional ultra-performance liquid chromatography with tandem mass spectrometry; 5-mdC, 5-methyl-2′ -deoxycytidine; 5-hmdC, 5-Hydroxymethyl-2'-deoxycytidin; 5-cadC, 5-carboxy-2′ -deoxycytidine; 5-fdC, 5-formyl-2′ -deoxycytidine; 5-hmdU, 5-hydroxymethyl-2′ -deoxyuridine;MeCP2, methyl CpG binding protein 2; EZH2, enhancer zeste homolog 2; PARP1, poly(ADP-ribose) polymerase 1; HCs, healthy controls.
